# Divergent transcriptomic signatures from putative mesenchymal stimuli in glioblastoma cells

**DOI:** 10.1038/s41417-023-00724-w

**Published:** 2024-02-09

**Authors:** William S. Hart, Paul J. Myers, Benjamin W. Purow, Matthew J. Lazzara

**Affiliations:** 1 Department of Chemical Engineering, University of Virginia, Charlottesville, VA 22903 USA; 2 Department of Neurology, University of Virginia, Charlottesville, VA 22903 USA; 3 Department of Biomedical Engineering, University of Virginia, Charlottesville, VA 22903 USA

**Keywords:** CNS cancer, Molecular biology

## Abstract

In glioblastoma, a mesenchymal phenotype is associated with especially poor patient outcomes. Various glioblastoma microenvironmental factors and therapeutic interventions are purported drivers of the mesenchymal transition, but the degree to which these cues promote the same mesenchymal transitions and the uniformity of those transitions, as defined by molecular subtyping systems, is unknown. Here, we investigate this question by analyzing publicly available patient data, surveying commonly measured transcripts for mesenchymal transitions in glioma-initiating cells (GIC), and performing next-generation RNA sequencing of GICs. Analysis of patient tumor data reveals that TGFβ, TNFα, and hypoxia signaling correlate with the mesenchymal subtype more than the proneural subtype. In cultured GICs, the microenvironment-relevant growth factors TGFβ and TNFα and the chemotherapeutic temozolomide promote expression of commonly measured mesenchymal transcripts. However, next-generation RNA sequencing reveals that growth factors and temozolomide broadly promote expression of both mesenchymal and proneural transcripts, in some cases with equal frequency. These results suggest that glioblastoma mesenchymal transitions do not occur as distinctly as in epithelial-derived cancers, at least as determined using common subtyping ontologies and measuring response to growth factors or chemotherapeutics. Further understanding of these issues may identify improved methods for pharmacologically targeting the mesenchymal phenotype in glioblastoma.

## Introduction

The ability of glioblastoma cells to adapt to different conditions allows them to survive in the inflammatory and hypoxic tumor microenvironment and to resist therapy, contributing to the universal lethality of these tumors. The bounds of adaptability in glioblastoma have been characterized independently in multiple studies using techniques to capture transcriptomic, proteomic, genomic, and patient survival data. Three predominant subtypes have emerged: mesenchymal, proneural, and classical [1–4]. The mesenchymal subtype is defined based on expression of genes including *CHI3L1*, *CD44*, *VEGF*, and *MERTK*, as well as genes associated with mesenchymal transitions in epithelial-derived cancers such as *FN1* and *VIM* [2, 5, 6]. Compared to other subtypes, mesenchymal tumors are more resistant to radiation and chemotherapy, angiogenic, invasive, and enriched in recurrent disease [1, 7–9]. Proneural, isocitrate dehydrogenase-mutant glioblastomas, which are associated with longer patient survival times, are characterized by expression of the neuronal genes *OLIG2* and *BCAN* and Notch pathway-related genes *DLL3, HEY2*, and *ASCL1* [1]. Classical glioblastoma tumors are also associated with longer survival than mesenchymal tumors, expression of transcripts including *PCNA* and *TOP2A*, and the EGFR variant III mutant [2]. In addition to gene expression differences, genetic and epigenetic factors may predispose a tumor to a particular subtype, further complicating the phenotypic landscape of glioblastoma [10–13]. While specific mutations can predispose a tumor to a particular subtype, such as predominantly mesenchymal tumors with *NF1* mutations, a hallmark of glioblastoma subtypes is their ability to change in response to external perturbations [2, 3].

Conversion among glioblastoma subtypes may occur in response to cues in the tumor microenvironment. Growth factors including TGFβ [12] and TNFα [13], which can be produced by transformed neoplastic cells or by tumor-associated microglia and macrophages [14], are reported to drive proneural-to-mesenchymal transition (PMT) via transcription factors including SMADs, ZEB1, NF-κB, STAT3, C/EBPβ, and TAZ [12, 13, 15, 16]. Similarly, the low oxygen tension (hypoxia) characteristic of poorly perfused glioblastoma tumors [17, 18] is reported to drive PMT through activation of C/EBPβ [18]. Glioblastoma cells utilize similar pathways when exposed to ionizing radiation or temozolomide, becoming resistant to therapy through the activation of C/EBPβ [11], PI3K/Akt [19], or NF-κB [20]. The studies that identified these PMT-promoting factors were largely performed by measuring a relatively small number of mesenchymal and proneural genes from cell lines in vitro or orthotopic tumor xenograft experiments. Given that glioma subtypes were defined using transcriptomic data from human tumors, these small-scale measurements may be subject to significant bias depending on the selection of particular subtype-specific genes. Understanding PMT regulation more broadly may aid in identifying treatments that preferentially target specific subtypes, as demonstrated for CDK4/6 [21] or EZH2 [22] inhibition for proneural glioblastomas and DGKα [23] or BMI-1 [22] inhibition for mesenchymal glioblastomas.

Here, we assessed glioblastoma cell responses to purported inducers of the mesenchymal transition using data primarily at the gene expression level. A small set of conditions was selected for study as PMT drivers, based on a literature survey and examination of publicly available human tumor data sets. Our analysis suggests that tumor microenvironment cues such as the growth factors TGFβ and TNFα may be involved in glioblastoma mesenchymal transitions but do not drive a uniform PMT. Instead, they appear to broadly promote the expression of both proneural and mesenchymal transcripts. The DNA alkylating agent temozolomide displays similar characteristics. Thus, interconversion among glioblastoma molecular subtypes is unlikely to occur in a distinct manner without the complex activation of multiple signaling pathways driven by the microenvironment that is present in tumor but not in vitro culture.

## Materials and methods

### Analysis of publicly available data

Glioblastoma transcriptome data from The Cancer Genome Atlas (TCGA) [10] were downloaded from cBioPortal. Glioblastoma proteome data (PDC000204) from the Clinical Proteomic Tumor Analysis Consortium (CPTAC) [4] were downloaded from the NCI Proteomic Data Commons. Single-cell RNA sequencing data from Neftel, et al. [3] were downloaded from the Broad Institute Single-Cell Portal.

Gene set variation analysis (GSVA) [24] was used to calculate sample-wise gene (or gene product) set enrichment scores for each data set. Hallmark gene sets were obtained from the Molecular Signatures Database. TCGA tumors were grouped based on subtype annotations provided in the dataset. To derive groups for the CPTAC proteome dataset, samples were clustered based on expression of Verhaak subtype genes [2] using the ‘M3C’ R package for Monte Carlo reference-based consensus clustering [25] and the partitioning around medoids (PAM) algorithm with 25 iterations. M3C consensus clustering yielded three groups, which were manually assigned as *mesenchymal*, *proneural*, or *other* based on Verhaak mesenchymal and proneural enrichment scores. For the single-cell transcriptome dataset [3], a UMAP (https://arxiv.org/abs/1802.03426) projection was generated using log-transformed transcripts per million (log_2_(TPM/10 + 1)) expression of genes described for CPTAC data clustering [2]. These data were previously filtered by Neftel, et al. to remove cells and genes with low expression [3]. The UMAP projection was created using a nearest neighbors setting of 30 and a minimum distance of 0.01. The samples projected in UMAP space were then clustered with ‘ConsensusClusterPlus’ [26] using Euclidean distance as the distance metric, 100 iterations, and the PAM algorithm, which yielded six optimal groups. Mesenchymal cells were identified as the cluster with the greatest enrichment of Verhaak, et al. mesenchymal gene scores and Neftel, et al. mesenchymal meta-module scores [2, 3]. Proneural cells were similarly identified as the cluster with the greatest enrichment of cells with proneural gene scores and OPC-like meta-module enrichment [2, 3]. Pearson correlations of GSVA scores and p-values were calculated using the *cor.test* function from the ‘stats’ R package.

### Cell culture

The glioma-initiating cell (GIC) lines G559 [21] and G816 [27] were obtained from Jakub Godlewski (Brigham and Women’s Hospital, Boston, MA) and Ichiro Nakano (University of Alabama), respectively. GIC lines were propagated in suspension culture in neurobasal medium with B27 and N-2 supplements, 0.25 mM L-glutamine, 100 units/mL penicillin, and 100 μg/mL streptomycin (all from Gibco), with 50 ng/mL human recombinant EGF and basic FGF (both growth factors from Peprotech). For experiments, GICs were plated adherently on cell culture wells coated with Matrigel basement membrane (Corning) diluted in 1 mL ice-cold PBS per well at a concentration of 76 μg/mL for 1 h at room temperature, followed by aspiration to remove excess PBS-Matrigel. All cell lines were confirmed mycoplasma-negative using a MycoAlert PLUS Detection Kit (Lonza). STR profiling to confirm human origin of G816 cells was conducted by the Genetic Resources Core Facility (RRID:SCR_018669), Johns Hopkins Department of Genetic Medicine, Baltimore, MD.

### Chemical reagents and growth factors

Temozolomide (Santa Cruz Biotechnology) was reconstituted in DMSO at 20 mg/mL, according to manufacturer recommendations. Recombinant human TGFβ1 and TNFα (both from Peprotech) were reconstituted in 10 mM citric acid (pH 3) and water, respectively.

### Immunofluorescence microscopy and automated image analysis

Cells were grown on Matrigel-coated 18-mm glass coverslips. After treatment, cells were fixed with Prefer Fixative (Anatech) for 10 min at room temperature, then permeabilized with 0.25% Triton-X 100 in PBS for 5 min. Primary antibody (N-cadherin, CST #14215 S) was diluted in Intercept Blocking Buffer (LI-COR) and incubated overnight at 4 °C in a humidified chamber. Coverslips were washed and incubated with Alexa Fluor secondary antibodies and Hoechst for 1 h at 37 °C. Coverslips were mounted on glass slides with ProLong Gold Antifade Mountant (Invitrogen).

Cells were imaged using a Zeiss Axiovert Observer Z1 fluorescence microscope using a 20× objective. Image analysis was performed using CellProfiler v3.1.8 (Broad Institute) to quantify cell area, form factor (4π × area/perimeter^2^ = 1 for a circle), and percent-touching (percentage of a cell’s boundary that is touching another cell’s boundary) using the nuclear stain to identify individual cells and N-cadherin to define cell boundaries.

### Quantitative reverse transcription PCR (RT-qPCR)

RNA was extracted from cells using the RNeasy kit (Qiagen) and reverse transcribed using the High-Capacity cDNA Reverse Transcription Kit (Applied Biosciences). RT-qPCR was performed using SYBR Green PCR Master Mix (Applied Biosystems) on a QuantStudio3 Real-Time PCR System (Applied Biosystems). Relative transcript abundance was determined using the comparative C_T_ method using *GAPDH* as a reference gene. RT-qPCR primer sequences are provided in Supplementary Table 1.

### RNA sequencing

RNA was extracted as described for RT-qPCR. Sample processing was performed by the UVA Genome Analysis and Technology Core (RRID:SCR_018883). RNA concentration was measured by Qubit assay, and quality control was performed using a TapeStation kit (Agilent). Library preparation was performed using the NEBNext Ultra II Directional RNA Library Prep Kit for Illumina, and mRNA sequencing was performed using a NextSeq 150-cycle high output kit (Illumina) and Illumina MiSeq 500 Sequencing System. Sequencing yielded at least 50 million reads for each sample, mapping to 17,791 genes. Transcript counts normalized by converting to log_2_ counts per million were used for all data analyses.

All analysis of RNA-seq data was performed in R. Differential expression analysis (DEA) was performed using ‘limma’ [28]. Statistical significance was calculated using moderated t-statistics, with adjusted p-values calculated using the Benjamini–Hochberg method to account for multiple comparisons testing. Normalized enrichment score and gene set overrepresentation analyses were performed using ‘clusterProfiler’ [29].

### Statistical analyses

For experiments where statistical significance was calculated, three biological replicates were measured. This sample size was based on a power analysis assuming a 50% change in gene expression with 10% error. Samples were compared using ANOVA with Tukey’s honestly significant difference or Games-Howell post-hoc testing for gene expression and cell morphology experiments, respectively. For conditions where only one comparison was needed, unpaired t-tests were used. Variance within each group was estimated by calculating standard deviation, and similar variances between groups were confirmed prior to statistical analysis. Statistical analyses were performed using R version 4.2.2.

## Results

### Patient tumor data support roles for TGFβ, TNFα, and hypoxia in promoting a mesenchymal glioblastoma phenotype

**Fig. 1:**
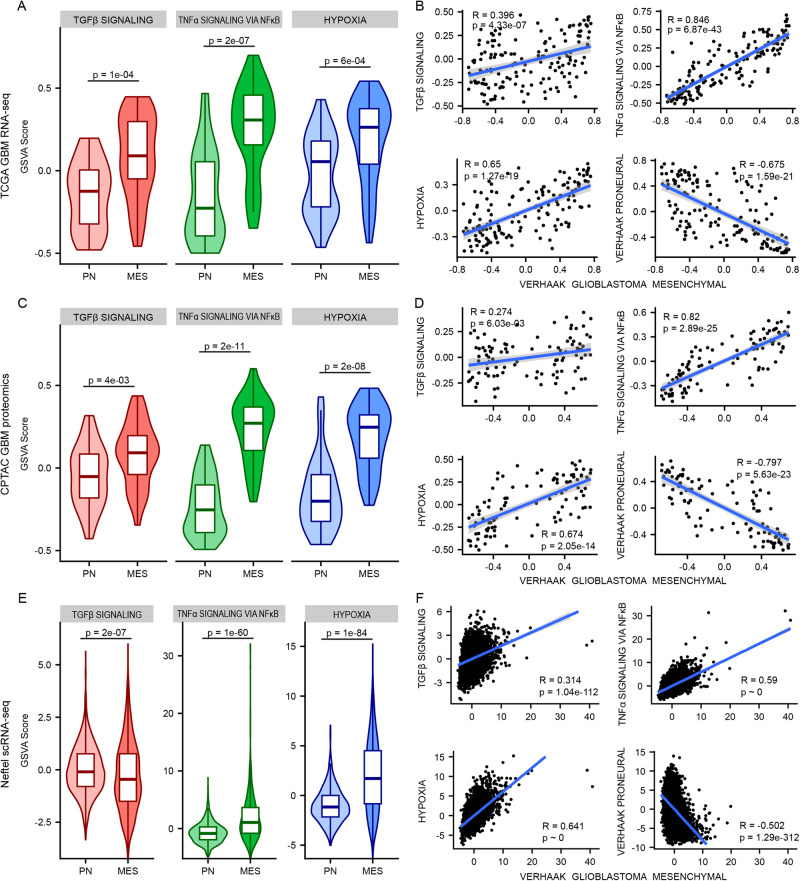
Analysis of publicly available data reveals that signaling in response to tumor microenvironment cues correlates with mesenchymal glioblastoma. **A** GSVA scores for Hallmark TGF-β signaling, TNFα signaling via NF-κB, and Hypoxia were calculated for each TCGA tumor categorized as proneural (PN, *n* = 29) or mesenchymal (MES, *n* = 49) [10]. Mann–Whitney rank-sum tests were used to calculate *p*-values. Boxplots represent the median and are bounded by the first and third quartiles. **B** GSVA scores for the pathways described in (**A**) and the Verhaak proneural gene set were used to calculate Pearson correlation coefficients (R) with GSVA scores for the Verhaak mesenchymal gene set for all TCGA glioblastoma tumors (*n* = 152) [10]. *p*-values for correlation coefficients were generated as described in Materials and Methods. **C** GSVA scores for the pathways described in (**A**) were calculated for each CPTAC glioblastoma tumor categorized as PN (*n* = 40) or MES (*n* = 35) [4]. Mann–Whitney rank-sum tests were used to calculate *p*-values. **D** GSVA scores for the pathways described in **(B)** and the Verhaak proneural gene set were used to calculate Pearson correlation coefficients with GSVA scores for the Verhaak mesenchymal gene set for all CPTAC glioblastoma tumors (*n* = 99) [4]. **E** GSVA scores for the pathways described in (**A**) were calculated for each Neftel, et al. [3] sample categorized as PN (*n* = 1128) or MES (*n* = 791). Mann–Whitney rank-sum tests were used to calculate *p*-values. **F** GSVA scores for the pathways described in **(B)** and the Verhaak proneural gene set were used to calculate Pearson correlation coefficients with GSVA scores for the Verhaak mesenchymal gene set for all malignant cells within the Neftel data set (*n* = 4916) [3]. *p* ~ 0 indicates a *p*-value calculated as equal to 0 due to the large sample size.

Because the literature suggests that TGFβ, TNFα, and hypoxia are common tumor microenvironment factors that drive PMT [12, 13, 17, 18], we first established their relevance in human tumors using publicly available patient data, beginning with TCGA bulk tumor transcriptomics [24]. Hallmark GSVA score enrichment in TGFβ, TNFα, and hypoxic signaling pathways was significantly greater in mesenchymal tumors relative to proneural tumors (Fig. 1A). Classical tumors were excluded from these analyses to isolate effects relevant to PMT. Additionally, the mesenchymal gene signature defined by Verhaak, et al. [2] significantly and positively correlated with gene signatures for TGFβ, TNFα, and hypoxic signaling and negatively with the proneural gene signature (Fig. 1B).

For CPTAC proteomic data [4], consensus clustering was first used to assign tumors to subtypes (see Materials and Methods), and the same GSVA approach described for TCGA data was then used to show again that TGFβ, TNFα, and hypoxic signaling are significantly upregulated in tumors identified as mesenchymal versus proneural (Fig. 1C). As in the bulk transcriptomic analysis, mesenchymal GSVA scores significantly correlated with Hallmark TGFβ, TNFα, and hypoxia signaling and negatively correlated with the proneural gene set (Fig. 1D).

To extend the analysis to account for intratumoral heterogeneity, single-cell transcriptomic data from Neftel, et al. [3] were also analyzed. UMAP projection and consensus clustering (Supplementary Fig. 1) were used to identify cells as mesenchymal or proneural, and comparisons were made for GSVA enrichment (Fig. 1E). TNFα and hypoxia pathways were significantly upregulated in mesenchymal compared to proneural cells, while TGFβ signaling was downregulated in mesenchymal cells. Mesenchymal enrichment correlated strongly with these pathways and negatively with proneural enrichment (Fig. 1F).

In aggregate, these analyses demonstrate correlations between TNFα, TGFβ, and hypoxia and the mesenchymal glioblastoma subtype, pointing to these microenvironmental factors as potential drivers of the mesenchymal phenotype in vivo, as previously described [13, 15, 30]. Across the three analyses performed, TNFα/NF-κB-related signaling was consistently more highly enriched in mesenchymal tumors and correlated with mesenchymal traits in cells compared to TGFβ signaling. This may indicate a more primary role for TNFα/NF-κB in driving PMT and a more supporting role for TGFβ. Importantly, the Verhaak mesenchymal gene set shares some genes with the Hallmark pathways examined here (Supplementary Fig. 2). The analysis presented in Fig. 1 utilized the complete gene sets, but further analysis demonstrated qualitatively consistent trends when overlapping genes were removed from GSVA calculations of Hallmark enrichment scores in all conditions except the CPTAC proteomic enrichment of Hallmark TGFβ signaling, which was not significantly different between subtypes (Supplementary Fig. 3).

### TGFβ, TNFα, and temozolomide promote expression of a subset of mesenchymal transcripts

**Fig. 2:**
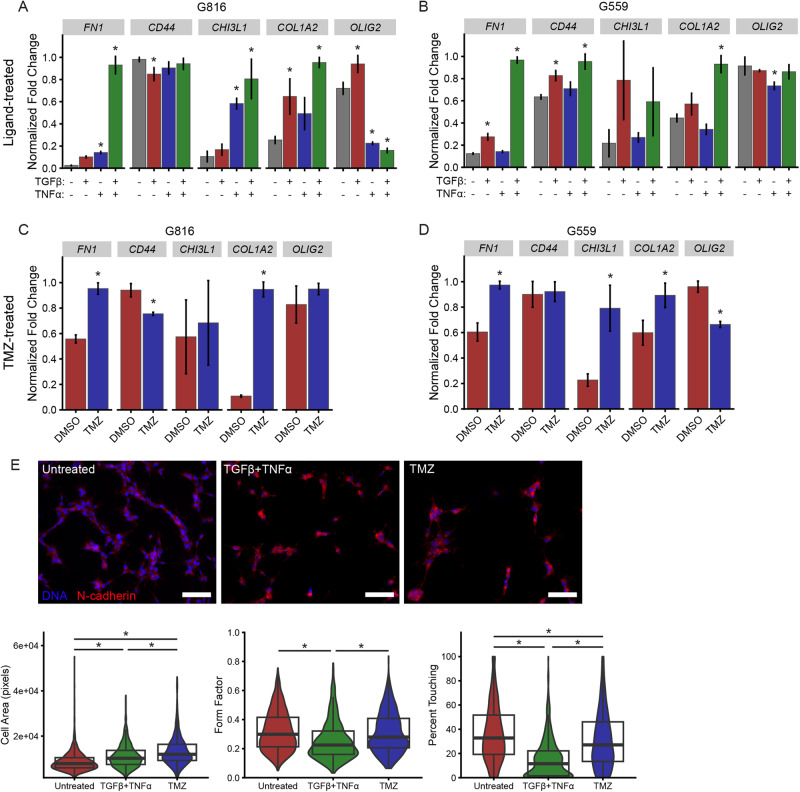
Growth factors and temozolomide promote expression of a subset of mesenchymal genes in glioma-initiating cells. G816 (**A**) or G559 (**B**) proneural GICs were plated on Matrigel and treated with 10 ng/mL TGFβ or TNFα, or both ligands, for 4 days. Expression of the mesenchymal markers *FN1*, *CD44*, *CHI3L1*, and *COL1A2* and the proneural marker *OLIG2* were measured by RT-qPCR. Error bars represent the mean ± standard deviation of three biological replicates. * indicates *p* < 0.05, as determined by one-way ANOVA and Tukey’s post-hoc test. Only significant differences with respect to the untreated condition are shown. G816 (**C**) or G559 (**D**) cells were plated on Matrigel and treated with DMSO (vehicle) or 100 μM temozolomide (TMZ) for 4 days, and the indicated transcripts were measured by RT-qPCR. Error bars represent the mean ± standard deviation of three biological replicates. * indicates *p* < 0.05 using two-tailed unpaired *t*-test. **E** G816 cells were plated on Matrigel-coated glass coverslips and treated with 10 ng/mL TGFβ + TNFα or 100 μM temozolomide (TMZ) for four days. Immunofluorescence microscopy was performed for N-cadherin to identify cell boundaries, and the indicated metrics (definitions in Materials and Methods) were extracted using automated image analysis, *n* = 3. * indicates *p* < 0.05 as determined by one-way ANOVA and Games-Howell post-hoc test. Scale bars = 100 μm.

To demonstrate that TGFβ and TNFα can promote mesenchymal gene expression, we treated two proneural GIC lines [21] with either or both recombinant ligands. Cells were plated on Matrigel to limit effects of ligand and oxygen gradients, which can arise in normal suspension spheroid culture of GICs when spheres grow to diameters >200 μm [31, 32]. In both cell lines, TGFβ and TNFα treatment for four days promoted the expression of several mesenchymal markers (Fig. 2A, B). The subset of markers we selected are commonly measured in small-scale investigations of PMT and associated with poor patient outcomes [13, 33]. TGFβ and TNFα both promoted mesenchymal marker expression as individual treatments and frequently cooperated when used in combination to drive larger changes in gene expression. Treatment of G816 cells with TGFβ + TNFα for six days at double the ligand concentration (20 ng/mL) did not qualitatively alter the expression of the markers measured except *CD44*, whose expression increased but only modestly by approximately 10% (Supplementary Fig. 4). Interestingly, TNFα was the only condition that led to significantly reduced expression of the proneural marker *OLIG2*. We also noted that GIC culture on Matrigel altered the expression of multiple PMT markers (Supplementary Fig. 5), indicating that Matrigel may promote some degree of PMT. Despite the effects of Matrigel on baseline GIC phenotype, exogenous ligand treatments still promoted mesenchymal gene expression.

We next investigated the role of temozolomide (TMZ), the frontline chemotherapeutic for glioblastoma, in promoting PMT. Previous reports have demonstrated that PMT occurs prior to glioblastoma recurrence, representing a potential mechanism by which primary tumors evade therapy [8, 9, 34]. The role of chemotherapy in driving phenotypic changes has typically been attributed to selection of chemoresistant cells, rather than to a direct effect of chemotherapy in promoting PMT [8, 34]. We found that a four-day treatment with a sub-lethal dose of TMZ (100 μM) promoted expression of the mesenchymal markers *FN1* and *COL1A2* in both GIC lines (Fig. 2C, D), consistent with the TMZ-driven upregulation of mesenchymal genes shown previously [35]. In addition to gene expression changes driven by TGFβ + TNFα and temozolomide, these treatments also altered cell morphology. TGFβ + TNFα-treated cells were less round with more protrusions, as indicated by a decreased form factor. Cell areas increased and cell-cell contacts decreased in response to both treatments (Fig. 2E). Decreases in form factor and cell-cell contact have been previously described as mesenchymal characteristics in vitro [12, 36].

The effect of hypoxia was also of interest due to its reported correlation with the mesenchymal phenotype in vivo (e.g., [3, 22] and Fig. 1). In the two GIC lines used in our experiments, however, hypoxia primarily led to an unexpected downregulation of mesenchymal markers (Supplementary Fig. 6). Hypoxia was therefore not investigated further. The unexpected effect of hypoxia we observed may arise from fundamental differences between the intact tumor microenvironment and in vitro cell culture setting. Indeed, the preponderance of evidence connecting hypoxia to PMT comes from in vivo studies [3, 17, 37, 38], where nonautonomous cancer cell effects (e.g., crosstalk with other cell types) are possible or where nutrient deprivation may promote glycolysis to drive PMT [39]. At least one study [40] has claimed that hypoxia drives PMT in vitro, but this study observed the upregulation of only two mesenchymal markers to support that claim.

### Transcriptomic analysis of GICs reveals divergent expression of subtype-specific genes in response to putative mesenchymal agonists

**Fig. 3:**
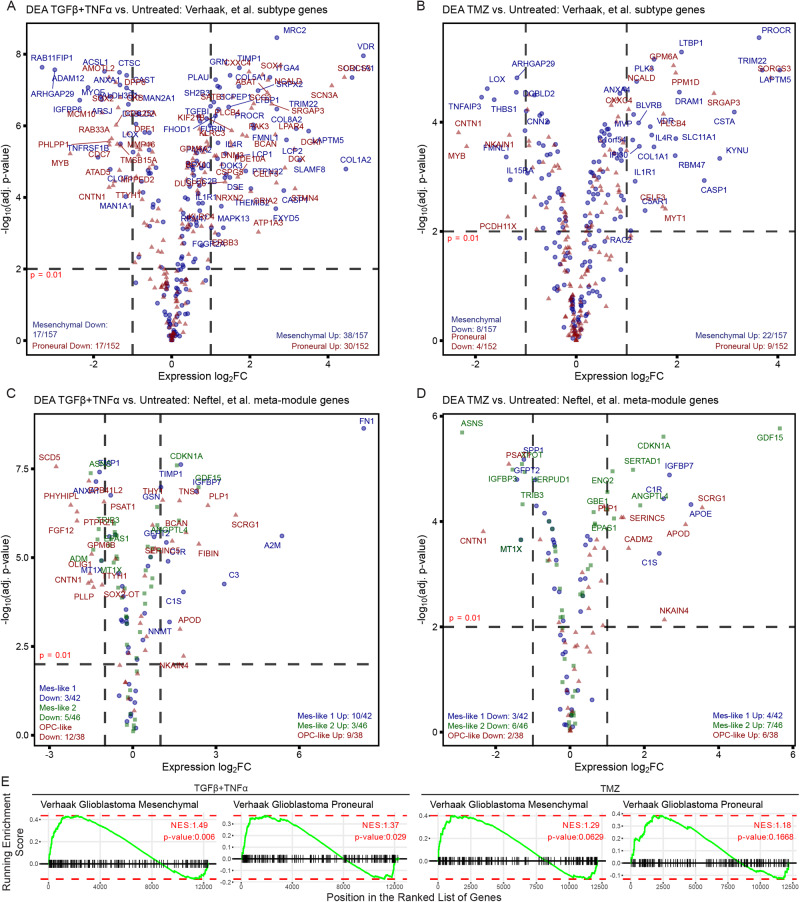
Purported PMT agonists broadly promote the expression of both proneural and mesenchymal genes. G816 cells grown on Matrigel were treated with (**A**) TGFβ + TNFα (10 ng/mL each) or (**B**) TMZ (100 μM) for 4 days. Volcano plots were generated by performing differential expression analyses comparing untreated cells to cells treated with TGFβ + TNFα or TMZ. Differential expression analysis (DEA) yielded the log_2_ fold-change (FC) of each transcript relative to the untreated condition. *p*-values were adjusted using Benjamini–Hochberg correction to account for multiple comparison testing. Genes from the Verhaak, et al. mesenchymal (blue circles) and proneural (red triangles) gene sets are shown to highlight the mixed phenotypic response to both TGFβ + TNFα and TMZ treatment. All analyses were performed using the expression of three independent replicates. **C**, **D** Data described in (**A**, **B**) were filtered instead for genes present in Neftel, et al. Mes-like 1 (blue circles), Mes-like 2 (green squares), and OPC-like (red triangles) meta-modules. **E** Normalized enrichment scores (NES) were calculated for each treatment relative to the untreated control for gene sets associated with the mesenchymal and proneural phenotypes. NES values and their statistical significance are displayed within plots of running enrichment scores.

To investigate the ability of the previously tested conditions to drive PMT using a more systematic and unbiased approach, we performed bulk RNA sequencing on one of the proneural GIC lines described in Fig. 2 treated for four days with TGFβ + TNFα or TMZ. TGFβ and TNFα were combined given the ability for these ligands to cooperate in driving mesenchymal gene expression (Fig. 2). Surprisingly, transcriptomic analysis revealed that growth factors simultaneously promoted and suppressed expression of both mesenchymal and proneural transcripts, as defined by Verhaak, et al. [2] (Fig. 3A). Temozolomide had a similar effect, though the numbers of significantly altered mesenchymal and proneural transcripts were lower (Fig. 3B). Analysis of genes defined by Neftel, et al. as mesenchymal-like hypoxia-independent (Mes-like 1), mesenchymal-like hypoxia-dependent (Mes-like 2), and the proneural analog oligodendrocyte progenitor cell (OPC-like) genes, similarly lacked substantial enrichment for either treatment [3] (Fig. 3C, D). Thus, neither treatment induced a clear, uniform phenotypic shift along the proneural-mesenchymal axis, as defined by established gene sets [2, 3].

To characterize mesenchymal and proneural gene expression further, we performed normalized enrichment score (NES) analysis for gene sets defined by Verhaak, et al. [2] (Fig. 3E). NES analysis for Verhaak gene sets [2] further showed that growth factors significantly enriched cells for both mesenchymal and proneural subtype genes, while TMZ treatment more highly enriched for mesenchymal genes than proneural genes, although not to a statistically significant degree. NES analysis supports the conclusion that the treatments did not induce uniform phenotypic shifts.

**Fig. 4:**
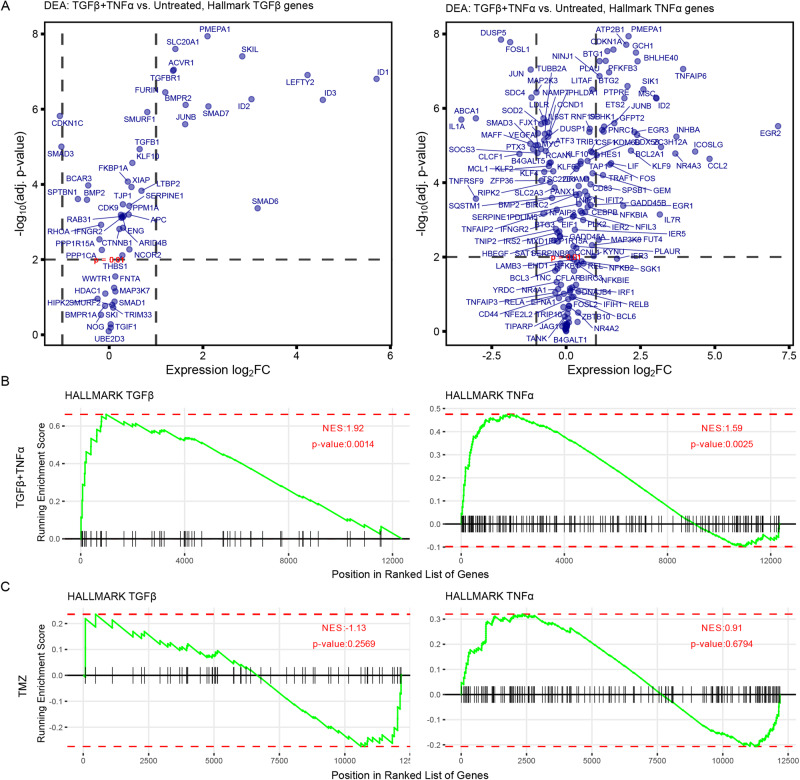
Exogenous TGFβ + TNFα promotes significant, anticipated transcriptomic enrichments. **A** Volcano plots were generated by performing differential expression analysis (DEA) on bulk RNA-seq data gathered from G816 cells (see Fig. 3) to compare TGFβ + TNFα-treated versus untreated samples. The log_2_ fold-changes (FC) of genes present in the Hallmark TGFβ and TNFα via NF-κB gene sets are shown. The Benjamini–Hochberg method was used to adjust *p*-values for multiple comparisons. **B**, **C** NES plots were generated for the Hallmark pathways in (**A**) for: (**B**) TGFβ + TNFα-treated samples and (**C**) TMZ-treated samples.

Differential expression and NES analyses using Hallmark TGFβ and TNFα gene sets for the ligand-treated samples confirmed that TGFβ and TNFα signaling were significantly enriched following treatment, as expected (Fig. 4A, B). The same analysis of TMZ-treated samples showed that TGFβ and TNFα signaling were not enriched following treatment with the drug, suggesting that gene expression changes induced by TMZ were driven independently of TGFβ and TNFα pathways (Fig. 4C).

**Fig. 5:**
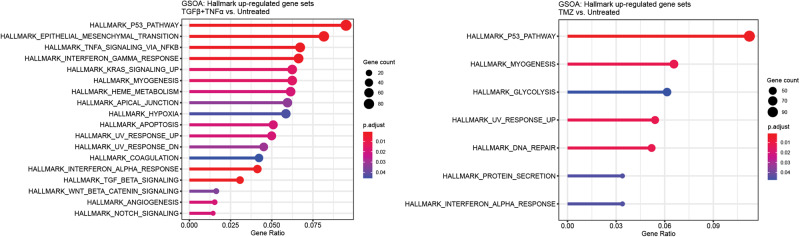
Purported drivers of PMT broadly promote gene expression consistent with epithelial-mesenchymal transition. Gene set overrepresentation analysis (GSOA) for Hallmark gene sets was performed on the RNA-seq samples for each treatment condition relative to untreated. Only pathways with significant (*p* < 0.05) overrepresentation are shown.

Finally, gene set overrepresentation analysis revealed signaling pathways preferentially activated by different treatment conditions (Fig. 5). Notably, growth factors promoted gene expression relating to epithelial-mesenchymal transition (EMT), β-catenin, and Notch, as well as TNFα and TGFβ signaling. Growth factors and TMZ both resulted in significantly upregulated genes related to p53 and interferon signaling, which promote EMT in multiple carcinomas [41–44]. Thus, while the treatment conditions we investigated do not drive a uniform and distinct PMT, they induce signaling associated with conventional EMT.

## Discussion

A central question addressed here is whether glioblastoma cells can transition from a proneural to a mesenchymal state in response to defined growth factor or chemotherapeutic treatments. While the specific conditions we tested are reported inducers of the mesenchymal state, prior work has typically assessed acute transitions using a small number of proteins or transcripts, far fewer than are used to define glioblastoma molecular subtypes. Assuming that a PMT defined as a shift in the preponderance of the markers included in subtyping systems is even possible, our findings raise the question of whether PMT requires the substantially more complex conditions reflective of the glioblastoma tumor microenvironment. Perhaps the best evidence that a PMT defined by established molecular subtypes can occur comes from in vivo studies using genetically barcoded, orthotopically implanted glioblastoma cells [3]. In those experiments, single-cell RNA sequencing revealed that cells of a single lineage can give rise to cells of each glioblastoma molecular subtype. That finding suggests that PMT based on subtype switching is possible, but the specific agonists, pathways, and timescale for that shift remain unknown.

Based on data presented here, it appears unlikely that simple cell culture conditions and small numbers of exogenous growth factors or drugs sufficiently recapitulate the complex signaling program necessary for a uniform PMT. Given that glioblastoma subtypes were identified through consensus clustering of tumors based on transcriptomic features, the subtype-defining genes broadly reflect the conditions of the tumor microenvironment and also the common genetic aberrations associated with the subtypes [2, 10]. While signaling associated with TGFβ, TNFα, and hypoxia correlate overall with the mesenchymal subtype, these correlations may primarily indicate that those conditions contribute to partial shifts to a mesenchymal state without a wholesale shift in subtype transcripts. This finding raises the interesting possibility that the diversity of microenvironmental factors present in glioblastomas may give rise to a diversity of mesenchymal states.

The lack of concordance among accepted glioblastoma subtyping strategies may exacerbate issues in using the subtyping features to monitor phenotypic shifts. For example, only three mesenchymal genes are shared [9] between the Phillips [1] and Verhaak [2] classifications. The more recent classification system developed by Neftel, et al. based on single-cell transcriptomics defined two distinct mesenchymal-like subtypes with similarly limited overlap with previous molecular subtyping systems [3] (Supplementary Fig. 7). Similar issues arise in molecular subtyping systems for pancreas cancer, for example, where there is virtually no overlap among gene sets for mesenchymal-like states and virtually no overlap of any of those gene sets with conventional EMT markers [45].

The field’s use of the term “mesenchymal” for one of the glioblastoma molecular subtypes may also encourage a potentially inappropriate tendency to view different subtypes, defined based on large numbers of transcriptomic features, as readily interconvertible in an analogous fashion to EMT in carcinoma cells, where acute exogeneous treatments of growth factors (e.g., TGFβ) drive clear phenotypic changes [46, 47]. While comparisons are frequently made between PMT and EMT, the naming of the mesenchymal glioblastoma subtype was based largely on the two markers CD44 and MERTK, with the latter being an unconventional mesenchymal marker [2]. Further, CD44 is most commonly associated with glioblastoma stemness, which is related but not identical to the mesenchymal phenotype [48]. Since glioblastoma molecular subtypes stratify patients for response to therapy [2], it is reasonable to hypothesize that drugging against specific subtypes could promote chemoresponse. While the rational identification of druggable signaling pathways that promote the mesenchymal phenotype could potentially be facilitated using network-level and multiscale computational models of cancer [49–51], our findings suggest that it may be challenging to identify treatment conditions that uniformly shift cells toward a glioblastoma mesenchymal state, which would be needed to generate the datasets required to develop such models. Therefore, it may be preferable simply to focus on specific phenotypes (e.g., migration or chemoresistance) or on subsets of the mesenchymal gene signature that play validated roles in regulating drug response, or to utilize alternative gene sets selected directly for their role in predicting therapeutic resistance [8]. For example, TGFβ may not drive a robust PMT, but TGFβ signaling strongly regulates response to temozolomide [30, 52]. Thus, identifying the specific genes altered by TGFβ in glioblastoma that control response to chemotherapy would provide an alternative approach for identifying drug targets independent of a specific assessment of PMT.

Following the example of EMT researchers [53], efforts should be made to formalize the nomenclature, definitions, and guidelines for studying mesenchymal transitions in glioblastoma, as driven in different contexts. Moreover, efforts should be made to distinguish transitions that occur in response to acute, well-defined treatments in cell culture model systems from true shifts among glioblastoma molecular subtypes. Based on the way molecular subtypes were originally defined, a robust definition of a PMT would include not only a shift in most subtype-defining transcripts but also an anticipated shift in the survival probabilities of animal models bearing proneural or mesenchymal tumors [2]. Isolated environmental effects (e.g., growth factors or hypoxia) experienced by glioblastoma cells in vitro or in vivo may drive changes in some of the subset-defining transcripts and alter acute responses to chemotherapies. Such effects are clearly worth elucidating, even if they do not represent a robust PMT. Whether the complex and heterogeneous conditions of the glioblastoma microenvironment are required to drive a bona fide PMT and whether such a transition is truly functionally distinct from the less uniform shifts observed as acute responses to isolated environment effects remains to be seen. Probing that issue is worthwhile though, as it may provide important new understanding for our ability to design durable treatments to slow glioblastoma progression.

### Supplementary information

Supplemental Material

## Data Availability

RT-qPCR C_T_ values and western blot raw images were submitted with the manuscript as “original data files.” RNA-seq data is available through the NCBI Gene Expression Omnibus (GEO #GSE229136). Publicly available data for the TCGA, CPTAC, and Neftel, et al. data sets are available from cBioPortal (https://www.cbioportal.org/study/summary?id=gbm_tcga_pub2013), NCI Proteomic Data commons (https://pdc.cancer.gov/pdc/study/PDC000204), and Broad Institute Single-Cell Portal (https://portals.broadinstitute.org/single_cell/study/SCP393/single-cell-rna-seq-of-adult-and-pediatric-glioblastoma), respectively.

## Code availability

All code used to generate figures and perform statistical analyses is available through GitHub (https://github.com/lazzaralab/Hart-et-al-GBM-PMT.git).

## References

[1] Phillips HS, Kharbanda S, Chen R, Forrest WF, Soriano RH, Wu TD (2006). Molecular subclasses of high-grade glioma predict prognosis, delineate a pattern of disease progression, and resemble stages in neurogenesis. Cancer Cell.

[2] Verhaak RG, Hoadley KA, Purdom E, Wang V, Qi Y, Wilkerson MD (2010). Integrated genomic analysis identifies clinically relevant subtypes of glioblastoma characterized by abnormalities in PDGFRA, IDH1, EGFR, and NF1. Cancer Cell.

[3] Neftel C, Laffy J, Filbin MG, Hara T, Shore ME, Rahme GJ (2019). An integrative model of cellular states, plasticity, and genetics for glioblastoma. Cell.

[4] Wang LB, Karpova A, Gritsenko MA, Kyle JE, Cao S, Li Y (2021). Proteogenomic and metabolomic characterization of human glioblastoma. Cancer Cell.

[5] Carro MS, Lim WK, Alvarez MJ, Bollo RJ, Zhao X, Snyder EY (2010). The transcriptional network for mesenchymal transformation of brain tumours. Nature.

[6] Mikheeva SA, Mikheev AM, Petit A, Beyer R, Oxford RG, Khorasani L (2010). TWIST1 promotes invasion through mesenchymal change in human glioblastoma. Mol Cancer.

[7] Mahabir R, Tanino M, Elmansuri A, Wang L, Kimura T, Itoh T (2014). Sustained elevation of Snail promotes glial-mesenchymal transition after irradiation in malignant glioma. Neuro Oncol.

[8] Segerman A, Niklasson M, Haglund C, Bergström T, Jarvius M, Xie Y (2016). Clonal variation in drug and radiation response among glioma-initiating cells is linked to proneural-mesenchymal transition. Cell Rep.

[9] Behnan J, Finocchiaro G, Hanna G (2019). The landscape of the mesenchymal signature in brain tumours. Brain.

[10] Brennan CW, Verhaak RGW, McKenna A, Campos B, Noushmehr H, Salama SR (2013). The somatic genomic landscape of glioblastoma. Cell.

[11] Minata M, Audia A, Shi J, Lu S, Bernstock J, Pavlyukov MS (2019). Phenotypic plasticity of invasive edge glioma stem-like cells in response to ionizing radiation. Cell Rep.

[12] Joseph JV, Conroy S, Tomar T, Eggens-Meijer E, Bhat K, Copray S (2014). TGF-β is an inducer of ZEB1-dependent mesenchymal transdifferentiation in glioblastoma that is associated with tumor invasion. Cell Death Dis.

[13] Bhat KPL, Balasubramaniyan V, Vaillant B, Ezhilarasan R, Hummelink K, Hollingsworth F (2013). Mesenchymal differentiation mediated by NF-κB promotes radiation resistance in glioblastoma. Cancer Cell.

[14] Ye X-Z, Xu S-L, Xin Y-H, Yu S-C, Ping Y-F, Chen L (2012). Tumor-associated microglia/macrophages enhance the invasion of glioma stem-like cells via TGF-β1 signaling pathway. J Immunol (Baltim, Md: 1950).

[15] Jiang Y, Zhou J, Hou D, Luo P, Gao H, Ma Y et al. Prosaposin is a biomarker of mesenchymal glioblastoma and regulates mesenchymal transition through the TGF‐β1/Smad signaling pathway. J Pathol. 2019. 10.1002/path.5278. 10.1002/path.5278 30953361

[16] Tamai S, Ichinose T, Tsutsui T, Tanaka S, Garaeva F, Sabit H, et al. Tumor Microenvironment in Glioma Invasion. Brain Sci. 2022;12.505. 10.3390/brainsci12040505 PMC9031400 35448036

[17] Piao Y, Liang J, Holmes L, Zurita AJ, Henry V, Heymach JV (2012). Glioblastoma resistance to anti-VEGF therapy is associated with myeloid cell infiltration, stem cell accumulation, and a mesenchymal phenotype. Neuro Oncol.

[18] Cooper LAD, Gutman DA, Chisolm C, Appin C, Kong J, Rong Y (2012). The tumor microenvironment strongly impacts master transcriptional regulators and gene expression class of glioblastoma. Am J Pathol.

[19] Tomar VS, Patil V, Somasundaram K (2020). Temozolomide induces activation of Wnt/β-catenin signaling in glioma cells via PI3K/Akt pathway: implications in glioma therapy. Cell Biol Toxicol.

[20] Aasland D, Gotzinger L, Hauck L, Berte N, Meyer J, Effenberger M (2019). Temozolomide induces senescence and repression of DNA repair pathways in glioblastoma cells via activation of ATR–Chk1, p21, and NF-kB. Cancer Res.

[21] Li M, Xiao A, Floyd D, Olmez I, Lee J, Godlewski J (2017). CDK4/6 inhibition is more active against the glioblastoma proneural subtype. Oncotarget.

[22] Jin X, Kim LJY, Wu Q, Wallace LC, Prager BC, Sanvoranart T (2017). Targeting glioma stem cells through combined BMI1 and EZH2 inhibition. Nat Med.

[23] Olmez I, Love S, Xiao A, Manigat L, Randolph P, McKenna BD (2018). Targeting the mesenchymal subtype in glioblastoma and other cancers via inhibition of diacylglycerol kinase alpha. Neuro Oncol.

[24] Hänzelmann S, Castelo R, Guinney J (2013). GSVA: gene set variation analysis for microarray and RNA-seq data. BMC Bioinforma.

[25] John CR, Watson D, Russ D, Goldmann K, Ehrenstein M, Pitzalis C (2020). M3C: Monte Carlo reference-based consensus clustering. Sci Rep.

[26] Wilkerson MD, Hayes DN (2010). ConsensusClusterPlus: a class discovery tool with confidence assessments and item tracking. Bioinformatics.

[27] Mao P, Joshi K, Li J, Kim S-H, Li P, Santana-Santos L (2013). Mesenchymal glioma stem cells are maintained by activated glycolytic metabolism involving aldehyde dehydrogenase 1A3. Proc Natl Acad Sci USA.

[28] Ritchie ME, Phipson B, Wu D, Hu Y, Law CW, Shi W (2015). limma powers differential expression analyses for RNA-sequencing and microarray studies. Nucleic Acids Res.

[29] Wu TZ, Hu EQ, Xu SB, Chen MJ, Guo PF, Dai ZH (2021). clusterProfiler 4.0: a universal enrichment tool for interpreting omics data. Innovation.

[30] Nie E, Jin X, Miao F, Yu T, Zhi T, Shi Z (2021). TGF-β1 modulates temozolomide resistance in glioblastoma via altered microRNA processing and elevated MGMT. Neuro Oncol.

[31] Park S, Avera AD, Kim Y (2022). Biomanufacturing of glioblastoma organoids exhibiting hierarchical and spatially organized tumor microenvironment via transdifferentiation. Biotechnol Bioeng.

[32] Jubelin C, Muñoz-Garcia J, Griscom L, Cochonneau D, Ollivier E, Heymann MF (2022). Three-dimensional in vitro culture models in oncology research. Cell Biosci.

[33] Colman H, Zhang L, Sulman EP, McDonald JM, Shooshtari NL, Rivera A (2010). A multigene predictor of outcome in glioblastoma. Neuro Oncol.

[34] Fedele M, Cerchia L, Pegoraro S, Sgarra R, Manfioletti G (2019). Proneural-mesenchymal transition: phenotypic plasticity to acquire multitherapy resistance in glioblastoma. Int J Mol Sci.

[35] Gao Z, Xu J, Fan Y, Qi Y, Wang S, Zhao S (2022). PDIA3P1 promotes Temozolomide resistance in glioblastoma by inhibiting C/EBPβ degradation to facilitate proneural-to-mesenchymal transition. J Exp Clin Cancer Res.

[36] Chandra A, Jahangiri A, Chen W, Nguyen AT, Yagnik G, Pereira MP (2020). Clonal ZEB1-driven mesenchymal transition promotes targetable oncologic antiangiogenic therapy resistance. Cancer Res.

[37] Piao Y, Liang J, Holmes L, Henry V, Sulman E, de Groot JF (2013). Acquired resistance to anti-VEGF therapy in glioblastoma is associated with a mesenchymal transition. Clin Cancer Res.

[38] Patel AP, Tirosh I, Trombetta JJ, Shalek AK, Gillespie SM, Wakimoto H (2014). Single-cell RNA-seq highlights intratumoral heterogeneity in primary glioblastoma. Science.

[39] Chen C, Shi Y, Li Y, He ZC, Zhou K, Zhang XN (2017). A glycolysis-based ten-gene signature correlates with the clinical outcome, molecular subtype and IDH1 mutation in glioblastoma. J Genet Genom.

[40] Joseph JV, Conroy S, Pavlov K, Sontakke P, Tomar T, Eggens-Meijer E (2015). Hypoxia enhances migration and invasion in glioblastoma by promoting a mesenchymal shift mediated by the HIF1α-ZEB1 axis. Cancer Lett.

[41] Imai D, Yoshizumi T, Okano S, Itoh S, Ikegami T, Harada N (2019). IFN-γ promotes epithelial-mesenchymal transition and the expression of PD-L1 in pancreatic cancer. J Surg Res.

[42] Yeh YH, Hsiao HF, Yeh YC, Chen TW, Li TK (2018). Inflammatory interferon activates HIF-1α-mediated epithelial-to-mesenchymal transition via PI3K/AKT/mTOR pathway. J Exp Clin Cancer Res.

[43] Chang CJ, Chao CH, Xia W, Yang JY, Xiong Y, Li CW (2011). p53 regulates epithelial-mesenchymal transition and stem cell properties through modulating miRNAs. Nat Cell Biol.

[44] Khan S, Mahalingam R, Sen S, Martinez-Ledesma E, Khan A, Gandy K, et al. Intrinsic interferon signaling regulates the cell death and mesenchymal phenotype of glioblastoma stem cells. Cancers (Basel). 2021;13.5284. 10.3390/cancers13215284 PMC8582372 34771447

[45] Brown BA, Myers PJ, Adair SJ, Pitarresi JR, Sah-Teli SK, Hart WS, et al. A histone methylation-MAPK signaling axis drives durable epithelial-mesenchymal transition in hypoxic pancreas cancer. bioRxiv 2022: 2022.2010.2019.512869. 10.1158/0008-5472.CAN-22-2945 PMC12032584 38471099

[46] Lu W, Kang Y (2019). Epithelial-mesenchymal plasticity in cancer progression and metastasis. Dev Cell.

[47] Buonato JM, Lan IS, Lazzara MJ (2015). EGF augments TGFbeta-induced epithelial-mesenchymal transition by promoting SHP2 binding to GAB1. J Cell Sci.

[48] Mooney KL, Choy W, Sidhu S, Pelargos P, Bui TT, Voth B (2016). The role of CD44 in glioblastoma multiforme. J Clin Neurosci.

[49] Day EK, Zhong Q, Purow B, Lazzara MJ (2021). Data-driven computational modeling identifies determinants of glioblastoma response to SHP2 inhibition. Cancer Res.

[50] Myers PJ, Lee SH, Lazzara MJ. Mechanistic and data-driven models of cell signaling: tools for fundamental discovery and rational design of therapy. Curr Opin Syst Biol. 2021;28.100349. 10.1016/j.coisb.2021.05.010 PMC9348571 35935921

[51] Warner HV, Sivakumar N, Peirce SM, Lazzara MJ (2019). Multiscale computational models of cancer. Curr Opin Biomed Eng.

[52] Joseph JV, Balasubramaniyan V, Walenkamp A, Kruyt FAE (2013). TGF-β as a therapeutic target in high grade gliomas—promises and challenges. Biochem Pharmacol.

[53] Yang J, Antin P, Berx G, Blanpain C, Brabletz T, Bronner M (2020). Guidelines and definitions for research on epithelial-mesenchymal transition. Nat Rev Mol Cell Biol.

