# Supplementary material for: Divergent transcriptomic signatures from putative mesenchymal stimuli in glioblastoma cells
Source: Cancer Gene Ther. 2024 Feb 9;31(6):851–60. doi: 10.1038/s41417-023-00724-w (PMC11192628; doi:10.1038/s41417-023-00724-w)
Supplement: Supplementary file 1 — Supplemental Material [file 41417_2023_724_MOESM1_ESM.docx]

**Supplemental Data**

Divergent transcriptomic signatures from putative mesenchymal stimuli in glioblastoma cells

Hart WS, et al.

**
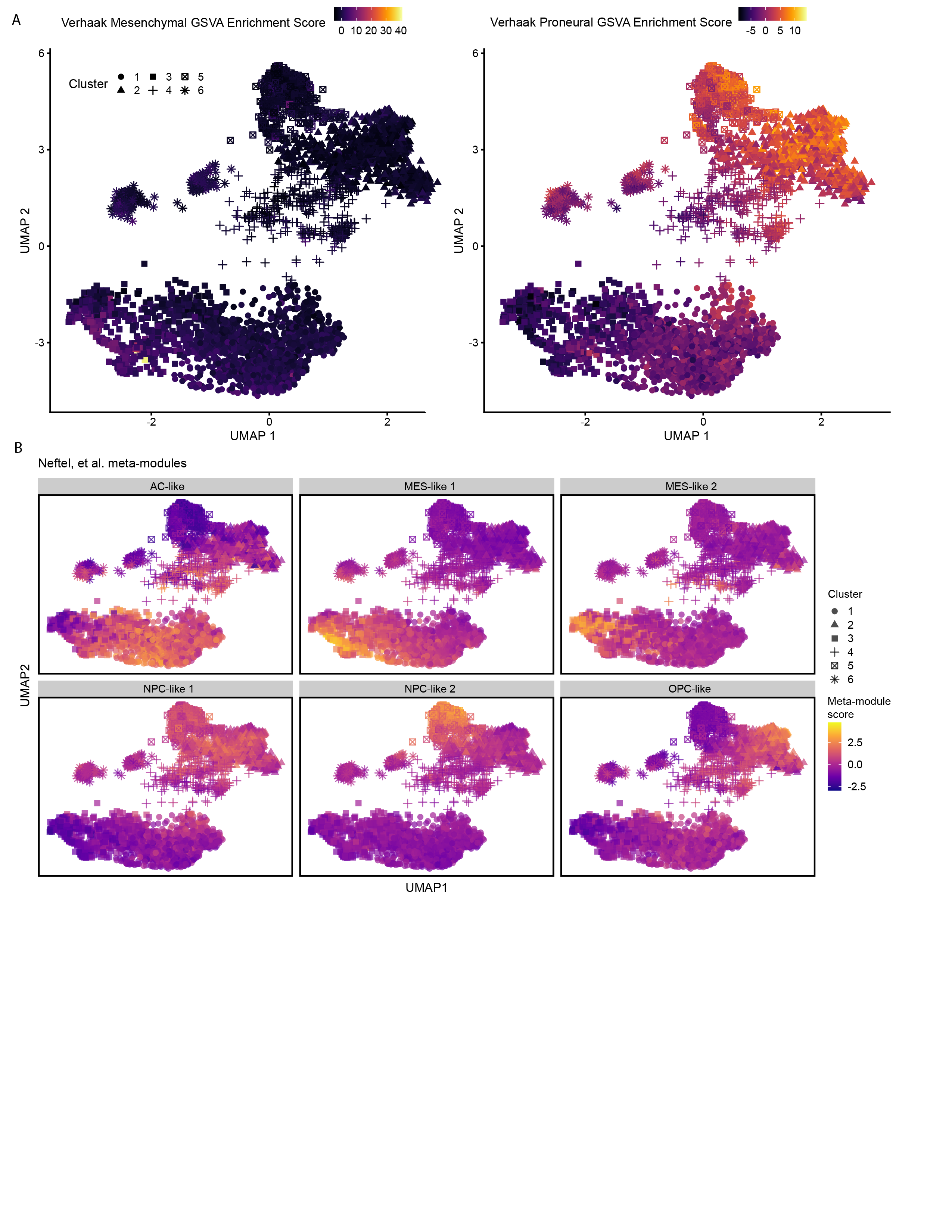
Supplemental Figure 1. UMAP projection and consensus clustering reduces data dimensionality and separates single cells into relevant subtypes.** Gene expression data from 4916 cells from 28 human glioblastoma tumors [1] were filtered for genes present in Verhaak mesenchymal, proneural, and classical gene sets [2] and used to generate a two-dimensional UMAP projection. Samples were categorized into one of six clusters using ConsensusClusterPlus [3], and clusters were labeled using **(A)** GSVA enrichment of mesenchymal and proneural gene sets, or **(B)** meta-module scores from Neftel, *et al*. [1]. Mesenchymal and proneural cells were assigned to clusters 3 and 2, respectively.


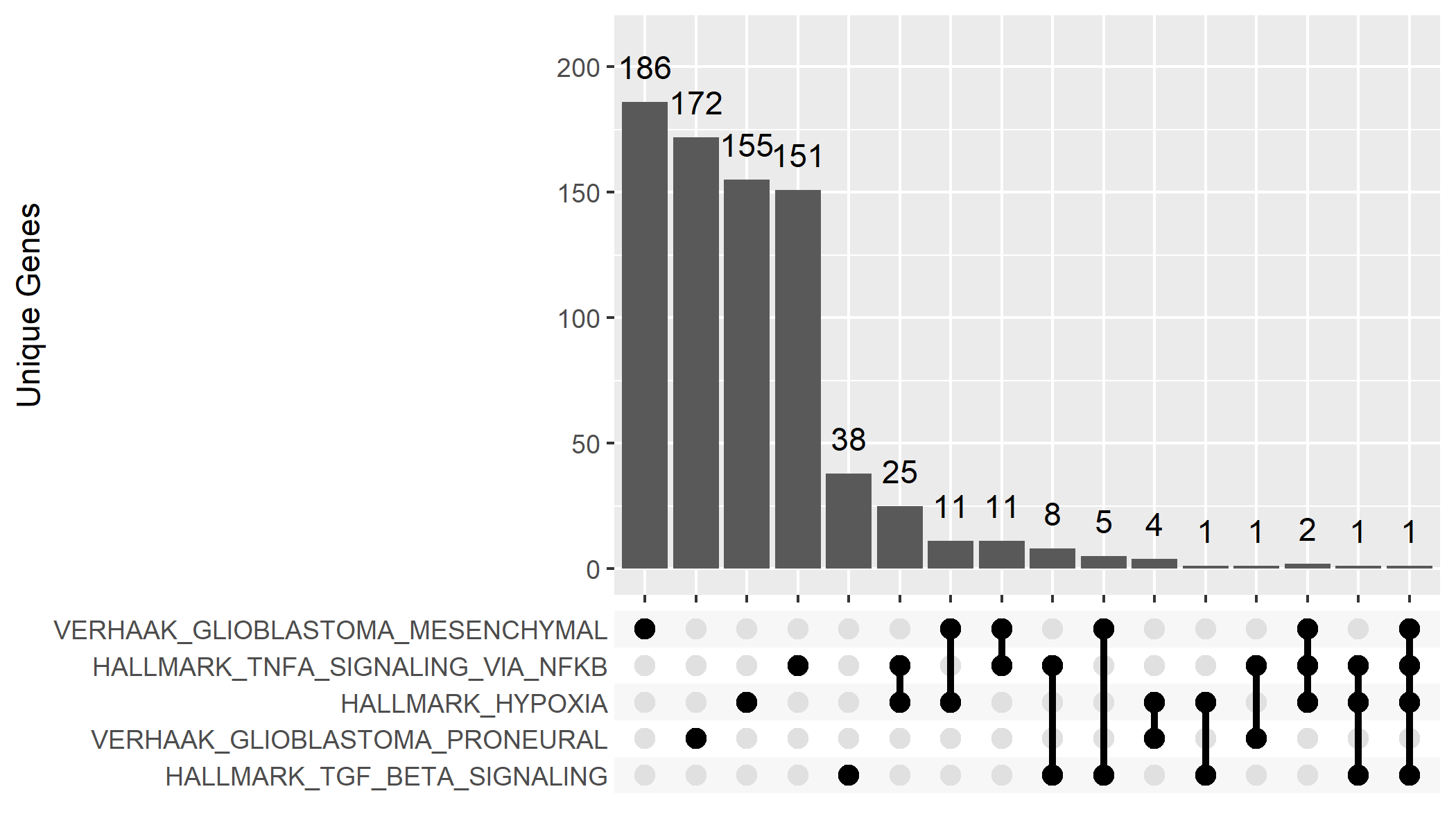


**Supplemental Figure 2.** **30 of 216 mesenchymal glioblastoma genes are shared with one or more relevant Hallmark signaling gene sets.** An UpSet plot shows the number of unique and overlapping genes among the glioblastoma mesenchymal and proneural subtypes [4] and the Hallmark TGFβ, TNFα, and hypoxia gene sets. The UpSet plot is formatted left to right to show the number of unique genes in each gene set, followed by genes that are shared among sets.

**
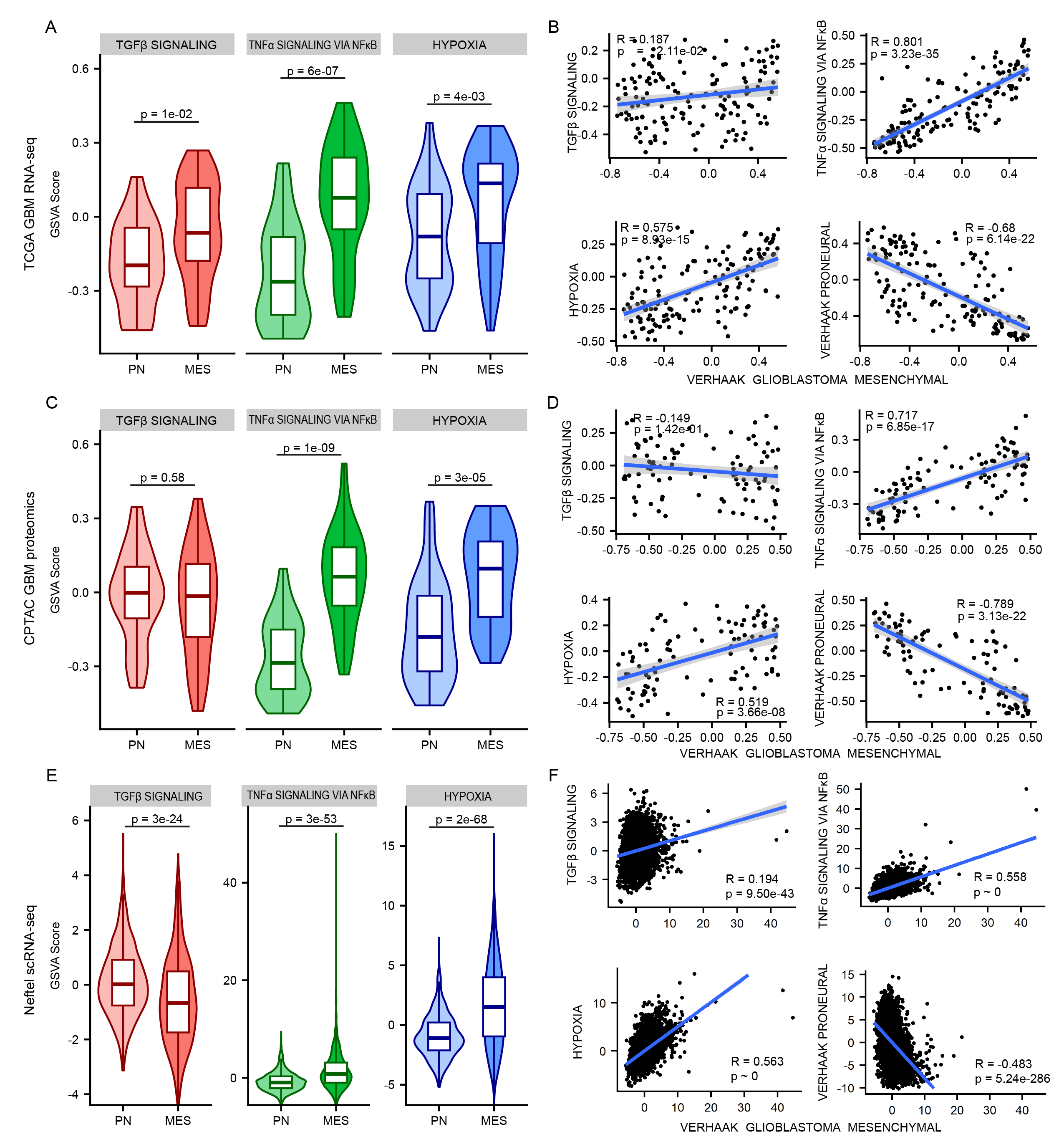
**

**Supplemental Figure 3. Concordance between mesenchymal subtype gene set enrichment and relevant Hallmark pathways remains largely unchanged after removing overlapping genes.** **(A)** GSVA scores for Hallmark TGFβ signaling, TNFα signaling via NF-κB, and Hypoxia signaling (all excluding genes shared with the glioblastoma mesenchymal gene set [4]) were calculated for each TCGA tumor categorized as proneural (PN, n = 29) or mesenchymal (MES, n = 49) [2]. Mann-Whitney rank-sum tests were used to calculate p-values. Boxplots represent median values bounded by the first and third quartiles. **(B)** GSVA scores for the pathways described in A) and the Verhaak proneural gene set were used to calculate Pearson correlation coefficients (R) with GSVA scores for the Verhaak mesenchymal gene set for all TCGA glioblastoma tumors (n = 152) [2]. p-values for correlation coefficients were generated as described in *Methods*. **(C)** GSVA scores for the pathways described in (A) were calculated for each CPTAC glioblastoma tumor categorized as PN (n = 40) or MES (n = 35) [5]. Mann-Whitney rank-sum tests were used to calculate p-values. **(D)** GSVA scores for the pathways described in B) and the Verhaak proneural gene set were used to calculate Pearson correlation coefficients with GSVA scores for the Verhaak mesenchymal gene set for all CPTAC glioblastoma tumors (n = 99) [5]. **(E)** GSVA scores for the pathways described in (A) were calculated for each Neftel, *et al*. [1] sample categorized as PN (n = 1527) or MES (n = 2381). Mann-Whitney rank-sum tests were used to calculate p-values. **(F)** GSVA scores for the pathways described in (B) and the Verhaak proneural gene set were used to calculate Pearson correlation coefficients with GSVA scores for the Verhaak mesenchymal gene set for all malignant cells within the Neftel data set (n = 4916) [1]. p ~ 0 indicates a p-value calculated as equal to 0 due to the large sample size.


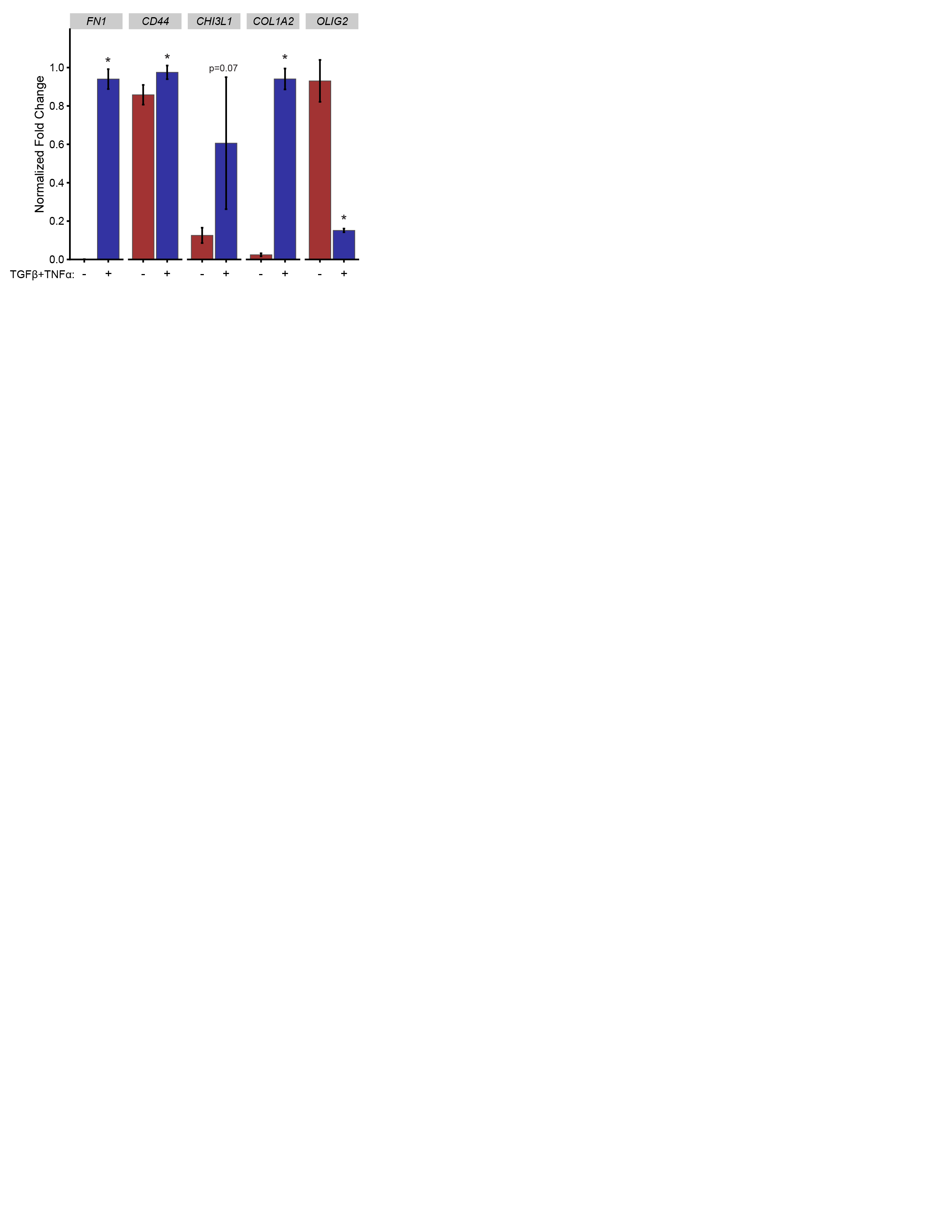


**Supplemental Figure 4. Increased growth factor concentrations for longer treatment times produces qualitatively similar results to lower treatment concentrations and durations.** G816 cells were plated on Matrigel and treated with 20 ng/mL TGFβ+TNFα for 6 days. Expression of the mesenchymal markers *FN1*, *CD44, CHI3L1*, and *COL1A2* and the proneural marker *OLIG2* were measured by RT-qPCR. Error bars represent the mean ± standard deviation of three biological replicates. * indicates p < 0.05 using a two-tailed unpaired t-test.


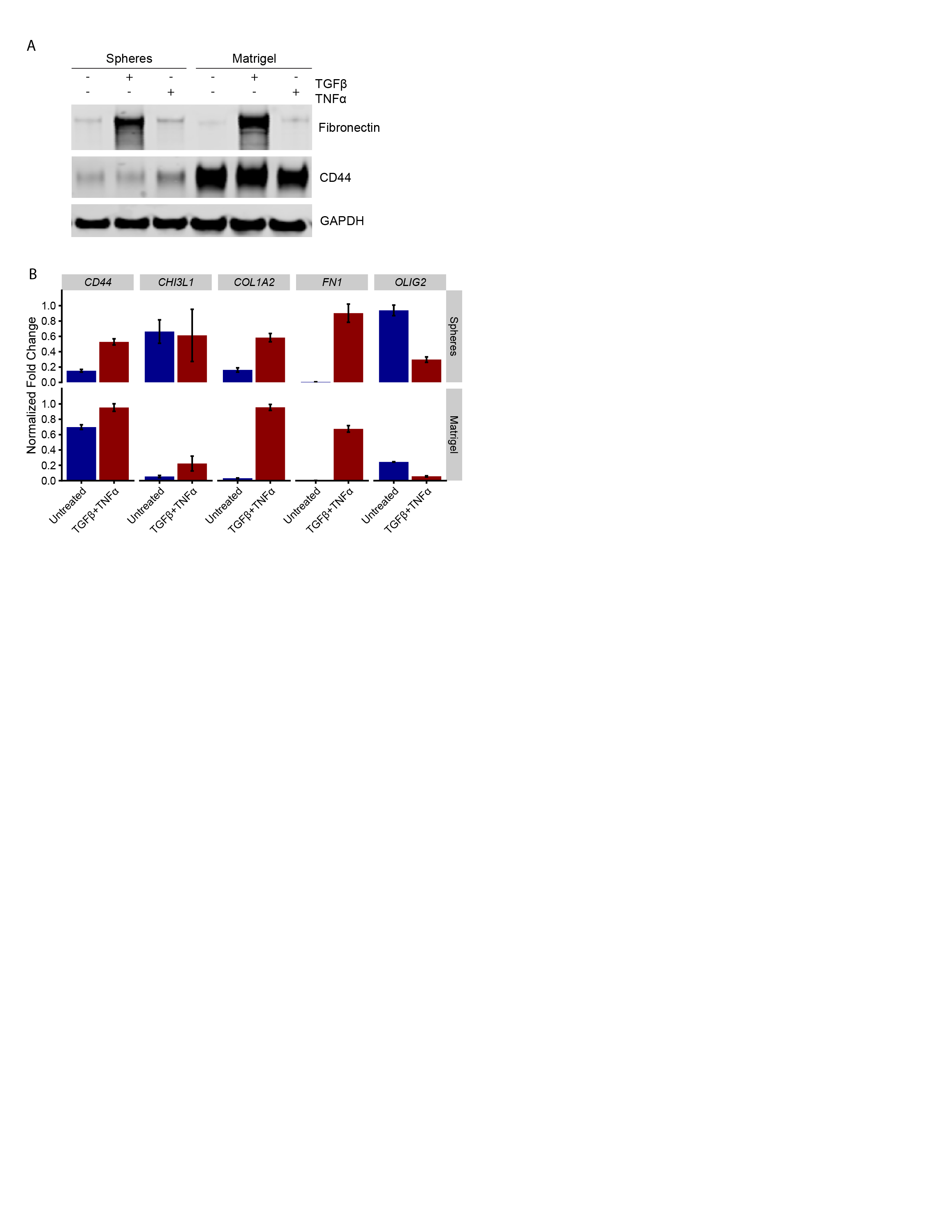


**Supplemental Figure 5. Culturing GICs on Matrigel alters mesenchymal and proneural gene expression.** G816 cells were cultured either as nonadherent spheres or on Matrigel. Cells were treated with 10 ng/mL TGFβ, TNFα, or both ligands as indicated for 4 days. Expression of the indicated **(A)** proteins or **(B)** transcripts were measured by western blot or RT-qPCR, respectively. The western blot image shown is representative of two independent replicates. Error bars represent the mean ± standard deviation of three biological replicates.

**
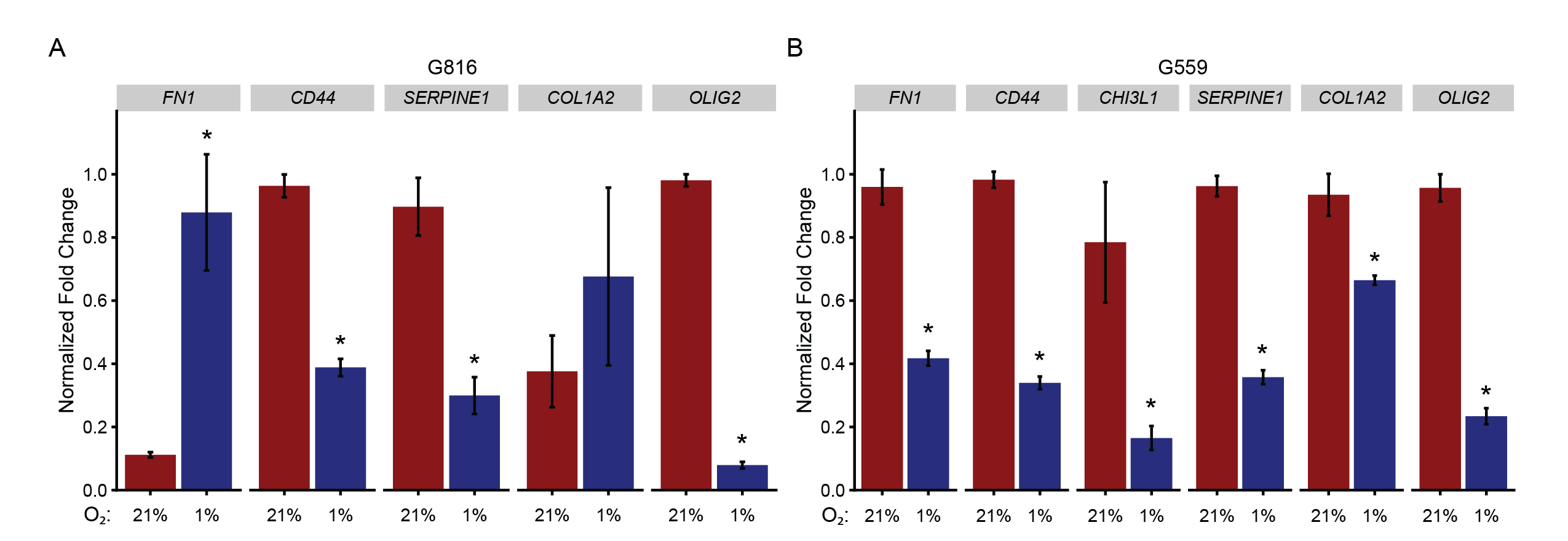
**

**Supplemental Figure 6. Hypoxia does not promote GIC PMT *in vitro*.** The GIC lines **(A)** G816 and **(B)** G559 were cultured in 21% or 1% O_2_ for 6 days, and gene expression of the indicated transcripts was assessed using RT-qPCR. *CHI3L1* is not shown for G816 cells due to low transcript abundance. Bars represent the mean ± standard deviation of three biological replicates. * indicates p < 0.01 using a two-tailed unpaired t-test.

**
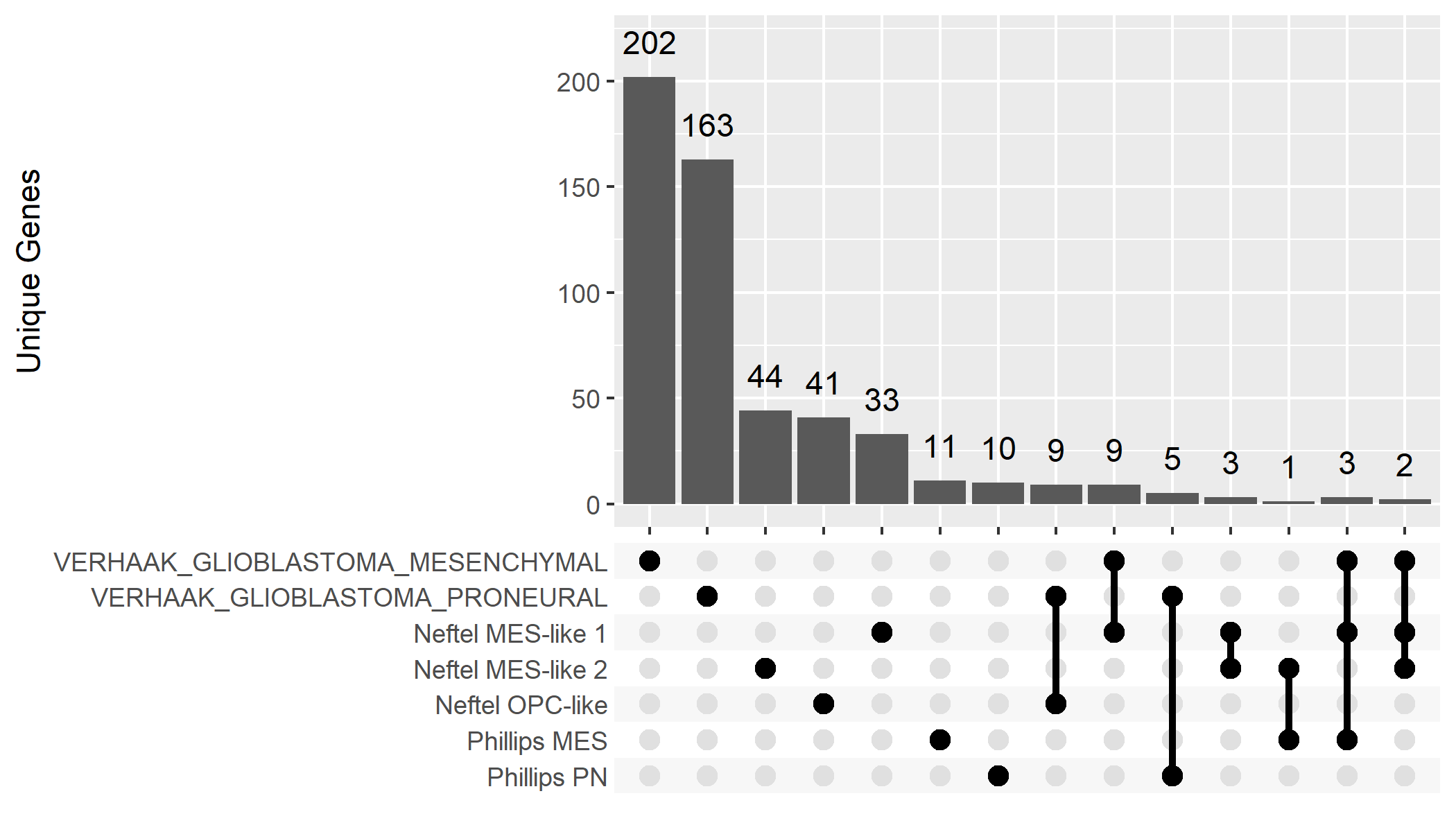
**

**Supplemental Figure 7. Gene sets describing mesenchymal and proneural subtypes of glioblastoma have low concordance.** Gene sets from supplemental references [4], [6], and [1] were compared via UpSet plot for numbers of shared genes. The UpSet plot was formatted left to right to show the number of unique genes in each gene set, followed by genes that are shared among sets.

**Supplemental Table 1. RT-qPCR primers.**

| **Gene** | **Forward** | **Reverse** |
| --- | --- | --- |
| *GAPDH* | GAGTCAACGGATTTGGTCGT | GACAAGCTTCCCGTTCTCAG |
| *FN1* | CAAGCCAGATGTCAGAAGC | GGATGGTGCATCAATGGCA |
| *CD44* | CTGATCATCTTGGCATCCCT | AGCTTTTTCTTCTGCCCACA |
| *CHI3L1* | CTCAAGAACAGGAACCCCAA | TCTGGGTGTTGGAGGCTATC |
| *COL1A2* | AAAACATCCCAGCCAAGAACTG | TCAAACTGGCTGCCAGCAT |
| *SERPINE1* | ATCGAGGTGAACGAGAGTGG | ACTGTTCCTGTGGGGTTGTG |
| *OLIG2* | AAATCGCATCCAGATTTTCG | GAAAAAGGTCATCGGGCTCT |

**Supplemental Methods**

*Western blotting*

Cell lysates were prepared in a standard cell lysis buffer (Invitrogen) supplemented with protease and phosphatase inhibitors (Millipore Sigma). Lysates were clarified by centrifugation at 20,000×g for 10 min at 4°C, and total protein concentrations were determined by micro-bicinchoninic assay (Thermo Fisher Scientific). Approximately 20 μg of denatured total protein was loaded per lane on 4-12% gradient polyacrylamide gels (Thermo Fisher Scientific) and transferred to 0.2 μm nitrocellulose membranes (Bio-Rad). Membranes were probed with antibodies and imaged on a LI-COR Odyssey CLx system. Antibodies used for western blotting included fibronectin (ab2413, Abcam), CD44 (#3570, CST), and GAPDH (sc-32233, Santa Cruz Biotechnology). Anti-mouse IgG IRDye700 conjugated (610-130-121) and anti-rabbit IgG DyLight800 (611-145-002) conjugated secondary antibodies were purchased from Rockland Immunochemicals.

*Hypoxic cell culture*

For experiments in hypoxia, cells were cultured in 1% oxygen using a Forma Steri-cycle i160 tri-gas incubator for six days with media replenished after three days.

*Visualization of gene set overlap*

UpSet plots were generated in R using the ‘ggupset’ package [7].

**SUPPLEMENTAL REFERENCES**

1 Neftel C, Laffy J, Filbin MG, Hara T, Shore ME, Rahme GJ *et al*. An Integrative Model of Cellular States, Plasticity, and Genetics for Glioblastoma. *Cell* 2019; 178: 835-849.e821.

2 Brennan CW, Verhaak RGW, McKenna A, Campos B, Noushmehr H, Salama SR *et al*. The somatic genomic landscape of glioblastoma. *Cell* 2013; 155: 462-462.

3 Wilkerson MD, Hayes DN. ConsensusClusterPlus: a class discovery tool with confidence assessments and item tracking. *Bioinformatics* 2010; 26: 1572-1573.

4 Verhaak RG, Hoadley KA, Purdom E, Wang V, Qi Y, Wilkerson MD *et al*. Integrated genomic analysis identifies clinically relevant subtypes of glioblastoma characterized by abnormalities in PDGFRA, IDH1, EGFR, and NF1. *Cancer Cell* 2010; 17: 98-110.

5 Wang LB, Karpova A, Gritsenko MA, Kyle JE, Cao S, Li Y *et al*. Proteogenomic and metabolomic characterization of human glioblastoma. *Cancer Cell* 2021; 39: 509-528.e520.

6 Phillips HS, Kharbanda S, Chen R, Forrest WF, Soriano RH, Wu TD *et al*. Molecular subclasses of high-grade glioma predict prognosis, delineate a pattern of disease progression, and resemble stages in neurogenesis. *Cancer Cell* 2006; 9: 157-173.

7 Lex A, Gehlenborg N, Strobelt H, Vuillemot R, Pfister H. UpSet: Visualization of Intersecting Sets. *IEEE Trans Vis Comput Graph* 2014; 20: 1983-1992.
